# Artificial Intelligence in 3D Aortic CTA Segmentation for Abdominal Aortic Aneurysm Management: A Narrative Review

**DOI:** 10.3390/jimaging12100479

**Published:** 2026-10-02

**Authors:** Leonardo Pasquetti, Edoardo Pasqui, Eleonora Cerbini, Manfredi Giuseppe Anzaldi, Giovanna Grasso, Bruno Gargiulo, Giuseppe Alba, Gianmarco de Donato

**Affiliations:** Vascular Surgery, Department of Medicine, Surgery and Neuroscience, University of Siena, 53100 Siena, Italy; edoardo.pasqui1@unisi.it (E.P.); eleonora.cerbini@student.unisi.it (E.C.); manfredi.anzaldi@student.unisi.it (M.G.A.); giovanna.grasso@student.unisi.it (G.G.); bruno.gargiulo@ao-siena.toscana.it (B.G.); giuseppe.alba@ao-siena.toscana.it (G.A.); dedonato@unisi.it (G.d.D.)

**Keywords:** abdominal aortic aneurysm, computed tomography angiography, aortic segmentation, artificial intelligence, endovascular aneurysm repair, three-dimensional reconstruction

## Abstract

Computed tomography angiography (CTA) is the reference imaging modality for diagnosis, endovascular planning, and surveillance of abdominal aortic aneurysms (AAAs), but conventional assessment remains time-consuming, operator-dependent, and limited by complex anatomy. This narrative review examines the role of artificial intelligence (AI) in three-dimensional aortic CTA segmentation for AAA management. The narrative synthesis drew on literature identified through PubMed/MEDLINE, Scopus and the Cochrane Library, with the original search covering January 2000 to July 2026. Ten primary studies from the existing bibliography were characterized in a study-level table, and reported Dice coefficients were displayed for nine studies. Current evidence indicates that AI-based segmentation, particularly using convolutional neural networks, U-Net-derived models, and nnU-Net frameworks, can improve the speed and reproducibility of aortic lumen, wall, thrombus, branch vessel, endograft, and aneurysm sac delineation. These techniques may support more accurate morphometric measurements, device sizing, procedural planning, quantitative surveillance, and the development of advanced imaging biomarkers. However, available studies remain heterogeneous in datasets, anatomical evaluation, imaging protocols, validation strategies, and reported performance metrics, limiting direct comparison and clinical generalizability. AI-assisted 3D segmentation represents a promising tool for transforming CTA from descriptive imaging into quantitative decision support, but robust multicenter validation, workflow integration, and expert supervision remain essential before widespread clinical adoption.

## 1. Introduction

### 1.1. Clinical Relevance of Aortic Disease

Aortic diseases include a wide spectrum of complex conditions, among which abdominal aortic aneurysms (AAAs) are of particular clinical significance. AAAs are associated with a high rate of morbidity and mortality, primarily due to the risk of rupture of the vascular wall. Indeed, the aortic wall becomes progressively thinner and more fragile as the dilation increases. Alterations in blood flow within the aneurysmal sac may also promote thrombus and embolus formation.

The risk of developing aortic disease increases with age, hypertension, and cigarette smoking [1,2]. AAA prevalence varies across populations and study periods. In a population-based screening program involving 65-year-old men in Stockholm, prevalence declined from 1.32% in 2010–2013 to 0.69% in 2020–2023 [1]. Moreover, despite increased risk factor control and improved screening, its rupture has a mortality rate of >80% [3]. It is an insidious disease, as in most cases, it is asymptomatic and its natural history is characterized by rupture. For this reason, early diagnosis, accurate risk stratification, longitudinal imaging surveillance, and timely treatment are essential components of modern vascular care. To achieve this, modern artificial intelligence (AI)-based three-dimensional computed tomography (3D CT) segmentation technologies play a pivotal role.

### 1.2. The Role of EVAR in Modern Aortic Treatment and Why Imaging Is Central in EVAR

Endovascular Aneurysm Repair (EVAR) is now the gold standard treatment for many patients with abdominal aortic aneurysm and suitable anatomy, with improved perioperative outcomes. The main advantages include lower perioperative mortality, less surgical trauma, and faster functional recovery with reduced hospitalization [2].

Within this endovascular context, radiological imaging plays a central role. Computed tomography angiography (CTA) is the gold standard for preoperative assessment, intervention planning, and long-term follow-up. Imaging is essential to accurately plan the procedure. Exact segmentation of the aortic lumen is essential to determine the lengths and diameters required for proper endograft sizing, limiting the risk of post-procedural complications such as endoleaks, which can result in continued expansion of the aneurysmal sac and eventual treatment failure. However, conventional analysis of CTA images brings significant challenges. Manual or semi-automatic interpretation of volumetric measurements is extremely time-consuming and subject to considerable inter-observer variability. Complex anatomies, tortuous vessels, or the presence of mural calcifications and thrombosis can make precise outlining of aortic contours difficult with traditional tools. Furthermore, the growing volume of data generated by modern CT scanners has drastically elevated the workload for radiologists and vascular surgeons, revealing the need for more efficient technological solutions [4,5].

### 1.3. Aim of the Review

The integration of AI and Deep Learning (DL) techniques into clinical practice represents a natural evolution to overcome the current limitations of manual image analysis. Recently, models based on convolutional neural networks (CNNs), such as the renowned U-Net architecture and its variants, have demonstrated excellent performance in the automated segmentation of organs and tissues, often yielding results superior to traditional computer vision methods in terms of speed and accuracy [6,7]. Automating 3D aortic segmentation would not only reduce the time required for preoperative planning but also ensure standardized measurements, providing the objective, reproducible indicators essential for precision medicine [8,9].

The primary objective of this narrative review is to analyze the current state of the art regarding the application of artificial intelligence to three-dimensional segmentation of aortic CTA in patients with AAA, with a specific focus on AAA management and its relevance to the clinical needs of vascular surgery. The review has an educational focus, explaining segmentation methods, anatomical targets, evaluation metrics and their relevance to vascular practice. The study-level synthesis is intended to contextualize the available evidence, without providing a comparative ranking of algorithms.

### 1.4. Search Strategy and Evidence Synthesis

A narrative rather than systematic format was adopted deliberately. Three-dimensional segmentation lacks a standardized definition and is reported across studies that differ substantially in design, segmentation modality and outcome definitions; this heterogeneity precludes a methodologically sound pooled estimate of a single effect size and instead favors an integrative, practice-oriented synthesis. During revision, the manuscript was assessed using the Scale for the Assessment of Narrative Review Articles (SANRA) [10]. The completed self-assessment is provided as Supplementary File S1. SANRA was applied to the review manuscript and was not used to assess the risk of bias of the primary studies. The original literature search covered publications from January 2000 to July 2026 in PubMed/MEDLINE, Scopus and the Cochrane Library. Controlled vocabulary (MeSH) and free-text terms were combined within and across the following concept blocks: (“aortic aneurysm” OR “aortic disease” OR “abdominal aortic aneurysm” OR AAA OR “aortic dissection”) AND (“computed tomography angiography” OR CTA OR “CT angiography”) AND (“three-dimensional segmentation” OR “3D segmentation” OR “medical image segmentation” OR “aortic segmentation”) AND (“artificial intelligence” OR “deep learning” OR “machine learning” OR “convolutional neural network” OR CNN OR “U-Net” OR “nnU-Net”) AND (“endovascular aneurysm repair” OR EVAR OR FEVAR OR BEVAR OR TEVAR OR “endovascular planning” OR “postoperative follow-up”), with Boolean operators adapted to the syntax of each database. The search was supplemented by manual screening of the reference lists of included articles, relevant society guidelines and recent systematic reviews. Records were screened by title and abstract, and potentially relevant full texts were assessed against the eligibility criteria. Study selection and data extraction were performed by L.P. and E.C. Disagreements were resolved through discussion, and when consensus was not reached, E.P. was consulted. Priority was given to studies evaluating artificial intelligence-based or semi-automated segmentation of aortic CTA datasets, with particular attention to segmentation accuracy, anatomical targets, clinical applicability, EVAR planning, postoperative follow-up and quantitative imaging biomarkers. Eligible sources comprised prospective and retrospective cohort studies, technical validation studies, imaging studies, registries, systematic reviews and meta-analyses; case reports were considered only when they illustrated specific technical or clinical applications not otherwise documented in larger series. Non-English articles and conference abstracts without subsequent full publication were excluded. The database retrieval identified 3576 PubMed records, 6604 Scopus records and 181 CENTRAL records; the same Cochrane Library search identified no Cochrane Reviews. Of 10,361 retrieved records, 3240 duplicates were removed, leaving 7121 unique records. These counts document identification and deduplication only. Systematic screening and full-text eligibility assessment of this reconstructed record set were not completed; therefore, numerical exclusions at these stages are not reported. The ten primary publications characterized in Supplementary Table S1 were selected from the narrative review bibliography for detailed descriptive synthesis and do not represent the endpoint of a completed systematic selection process. Other references support the clinical background, methodological explanations and conceptual discussion.

Given the heterogeneity of study designs, segmentation targets, imaging protocols, artificial intelligence architectures and reported performance metrics, the evidence was synthesized narratively and organized thematically around the main domains of this review:

- Analyzing the technical concept of 3D segmentation, the specific anatomical structures (lumen, wall, intraluminal thrombus, collateral branches) that can be isolated within CTA datasets and its role in EVAR
- Discussing current evidence on the efficacy of Deep Learning algorithms applied to the aorta, comparing different approaches (2D vs. 3D CNNs) and the results achieved in terms of performance metrics such as the Dice similarity coefficient (DSC).
- Highlighting the clinical benefits of automation, including reduced analysis times, personalized decision support, and the extraction of advanced quantitative biomarkers.
- Examining current limitations and barriers, such as the need for large, expert-annotated training datasets, model generalizability issues, and challenges related to AI explainability (Explainable AI).
- Explore future perspectives, including “human-in-the-loop” models, the use of aortic digital twins, and the integration of hemodynamic simulations based on segmented geometry to predict treatment outcomes.

Within each domain, priority was given to the most robust, clinically relevant and recent data, while conflicting findings were presented and discussed rather than pooled statistically. A formal quantitative meta-analysis and structured risk-of-bias assessment were not undertaken, consistent with the narrative design. Ten primary publications from the existing bibliography were selected for detailed characterization in Supplementary Table S1, provided as supplementary material. Background and methodological references were retained separately for the narrative discussion. Studies covering broader aortic regions or general CT datasets were identified as such and were not treated as direct evidence of infrarenal AAA performance. For each study, the table summarizes study design, cohort size, training/validation/test allocation, external evaluation status, aortic segment, target structures, architecture, segmentation metrics, errors in clinical measurements, and data and code availability. NA denotes information not reported, not reliably ascertainable from accessible sources, or not applicable. Reported mean Dice similarity coefficients (DSC) were plotted by target structure for the nine studies reporting this metric (Figure S1), without pooling or statistical comparisons.

## 2. Aortic Imaging for EVAR: The Role of Computed Tomography Angiography

### 2.1. Why CTA Is the Reference Standard

CTA is currently the primary and irreplaceable imaging technique for the evaluation, clinical management, and long-term monitoring of patients with aortic diseases, particularly AAA [11].

Its status as the gold standard stems from its ability to provide a detailed, three-dimensional representation of the entire vascular anatomy, allowing visualization not only of the aortic lumen but also of the vessel wall, the presence of mural thrombi, and the relationship of the aorta to surrounding vital organs [12].

In clinical practice, the quantitative analysis of CTA data is considered essential for accurately identifying pathologies and, above all, for performing rigorous preoperative planning. Compared to other modalities such as ultrasound, which remains useful for initial screening, or magnetic resonance imaging (MRI), which can provide advanced hemodynamic information but is less accessible and more expensive, CTA offers superior spatial resolution, which is essential for the precise sizing of endovascular prostheses [11].

### 2.2. Key CTA Features for EVAR Planning

The primary goal of CTA prior to an EVAR procedure is to provide an anatomical “map” that enables stent-graft sizing. Accurate segmentation of the lumen is the first step toward obtaining robust and reproducible morphological measurements. Specifically, the CTA must provide the surgeon with the following critical information:

1. Diameters and lengths: It is necessary to accurately measure the diameter of the aneurysmal neck (the portion of the healthy aorta above the aneurysm) to ensure a tight seal of the endoprosthesis [4,11].
2. Morphology of the aorta and iliac access vessels: It is crucial to evaluate the calcific and thrombotic burden. For example, calcified iliac arteries may make endoprosthesis advancement and deployment more difficult, while a calcified or thrombotic aortic neck may result in incomplete or unpredictable endograft expansion at the sealing zone.
3. Relationship with branch vessels: CTA allows for the localization of the origins of the renal arteries, the celiac trunk, and the superior mesenteric artery. The distance between these branches and the aneurysmal sac determines whether the patient is a candidate for standard EVAR or requires more complex fenestrated or branched stents.
4. Involvement of the iliac arteries: The extent of the aneurysm toward the common iliac arteries and the condition of the femoral access vessels are critical factors in selecting the catheter insertion route and positioning the “branches” of the stent graft.
5. Analysis of tortuosity: CTA allows for the assessment of the tortuosity of the iliac vessels and the aortic arch, a factor that can make it technically difficult to advance the guidewires and deploy the stent graft [4].

### 2.3. Limits of Conventional CTA Reading

Although CTA is the gold standard, conventional CTA analysis has significant limitations that the introduction of AI-based 3D segmentation aims to address:

- Inter-observer variability: Manual or semi-automatic interpretation of CTA images depends heavily on the operator’s experience and the quality of the software tools used. Different experts may interpret the boundaries of an aneurysm differently, leading to discrepancies in measurements that can influence the choice of stent size.
- Inaccuracy of 2D measurements: Measurements taken on two-dimensional axial slices are often imprecise if the aorta is tortuous or angled. Without a three-dimensional reference to the vessel’s centerline, diameters may be overestimated, since the detected cross-section is not perpendicular to the axis of flow [4].
- Anatomical complexity: In the presence of intraluminal thrombus (ILT), massive calcifications, or complex geometries (such as in true- and false-lumen dissections), manual contour delineation becomes an extremely arduous and error-prone task. In particular, distinguishing the true lumen from the false lumen in type B dissections requires a slice-by-slice analysis that is technically time-consuming [6].
- Workload and time: Manual annotation can be highly time-consuming, particularly when multiple structures must be delineated across thin-slice CTA volumes [13]. This time-consuming process makes detailed volumetric analysis difficult to apply on a large scale in routine clinical practice without the support of automated tools.

## 3. From CTA to 3D Modeling: What Segmentation Means

In this context, medical image segmentation extends beyond conventional anatomical visualization by transforming CTA datasets into patient-specific three-dimensional models that can be quantitatively analyzed and integrated into advanced computational and clinical workflows.

### 3.1. Definition of Medical Image Segmentation

Medical image segmentation is the process of partitioning a radiological dataset into distinct anatomical regions, enabling the identification, isolation, and quantitative analysis of individual anatomical structures. In the context of aortic CTA, segmentation enables the differentiation of the vascular lumen, aneurysm wall, ILT, calcifications, and branch vessels from the surrounding tissues. Through voxel-wise classification, where voxels represent the three-dimensional volumetric elements that constitute a CT dataset, corresponding to the three-dimensional equivalent of pixels, segmentation generates dedicated digital masks that can subsequently be converted into two-dimensional contours, three-dimensional surfaces, polygonal meshes, or complete patient-specific 3D models. These models provide the foundation for a wide range of advanced applications, including procedural planning, biomechanical simulations, computational fluid dynamics (CFD), and patient-specific three-dimensional (3D) printing [14].

**Figure 1:**
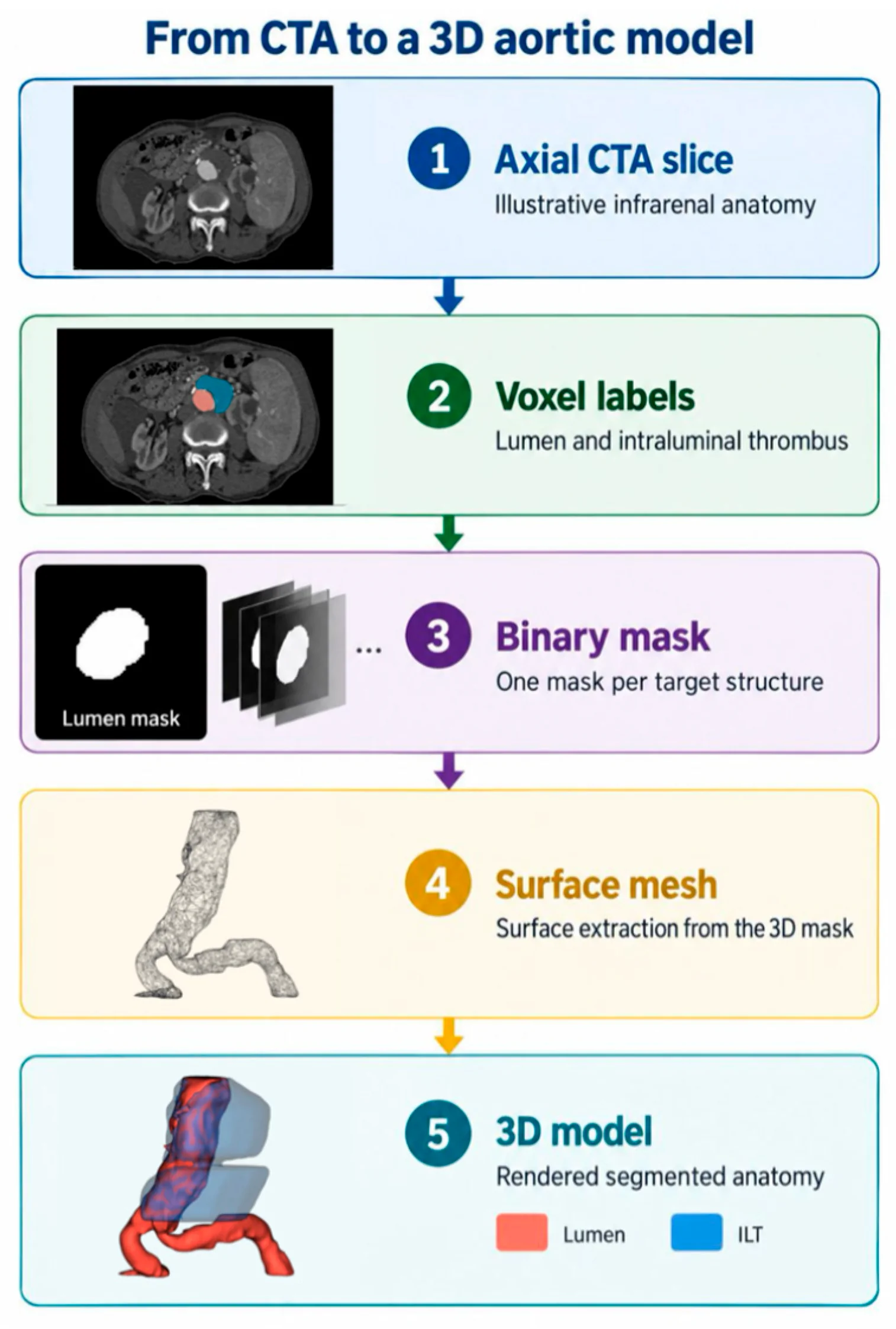
Stepwise workflow for three-dimensional CTA segmentation of an infrarenal abdominal aortic aneurysm using 3D Slicer (version 5.8.1; open-source software). The sequence shows an axial CTA slice, voxel-wise labeling of the contrast-filled lumen and intraluminal thrombus, binary-mask generation, surface-mesh extraction, and the final patient-specific three-dimensional model. The red region represents the aortic lumen, whereas the semi-transparent blue region represents the intraluminal thrombus (CTA: computed tomography angiography; ILT: intraluminal thrombus). The CTA images and derived segmentations were prepared by the authors from a clinical examination acquired during routine care at Azienda Ospedaliero-Universitaria Senese, Siena, Italy. The displayed material was de-identified and is presented solely to illustrate the segmentation workflow.

The overall workflow of CTA-based medical image segmentation, from image acquisition to patient-specific quantitative analysis and clinical decision-making, is summarized in Figure 1.

It is important to distinguish three-dimensional visualization from true quantitative segmentation. Conventional post-processing techniques, including multiplanar reconstruction (MPR), maximum intensity projection (MIP), and volume rendering (VR), provide increasingly realistic representations of vascular anatomy, thereby improving the spatial understanding of complex anatomical structures. In particular, volume rendering assigns color and opacity to voxels according to their attenuation values, allowing direct visualization of internal anatomical structures without explicit tissue classification.

However, these techniques primarily provide enhanced visual representations of the original imaging dataset and do not enable the quantitative isolation of individual anatomical components. In contrast, segmentation assigns a specific anatomical label to each voxel, generating digital structures that are directly measurable and quantitatively analyzable. For example, whereas conventional three-dimensional reconstruction can accurately depict the overall morphology of an AAA, precise segmentation enables quantitative assessment of the maximum aneurysm diameter, aneurysm volume, intraluminal thrombus volume and distribution, calcification burden, vascular tortuosity, and the spatial orientation of branch vessels. These quantitative parameters constitute the basis for patient-specific preoperative planning, biomechanical modeling, and the development of precision medicine workflows.

From a historical perspective, the earliest applications of medical image segmentation emerged during the 1980s, primarily in neuroradiology and oncology, where accurate delineation of tumors and adjacent anatomical structures was essential for neurosurgical and radiotherapy planning. During the same period, early segmentation algorithms were also applied to abdominal imaging for the localization and characterization of focal hepatic lesions, supporting liver resection planning and therapeutic monitoring. Throughout the 1990s, continuous advances in imaging technology and image-processing methodologies progressively expanded the clinical applications of segmentation across multiple medical specialties. The first applications in cardiovascular imaging were reported in the early 2000s, initially focusing on vascular lumen segmentation and automatic extraction of the aortic centerline to facilitate endovascular procedure planning.

Initially, segmentation was performed manually through slice-by-slice delineation of anatomical structures by experienced operators. Although highly accurate, this approach was time-consuming and subject to considerable inter-operator variability. Subsequently, semi-automatic techniques based on attenuation thresholding, automatic vessel boundary detection, and centerline extraction were introduced to improve processing speed, reproducibility, and standardization of image analysis.

More recently, the introduction of AI and deep learning techniques has further accelerated the evolution of medical image segmentation. In particular, CNNs, a class of algorithms specifically designed to automatically learn anatomical features directly from imaging data, have demonstrated excellent performance in the automated segmentation of vascular structures from CTA datasets [6]. Unlike conventional methods based on predefined rules or intensity thresholds, these models can recognize complex anatomical patterns and adapt to the substantial morphological variability observed in aortic diseases.

The earliest vascular surgery applications of deep learning primarily focused on automatic aortic segmentation within multiorgan segmentation frameworks. Subsequently, dedicated algorithms were developed to identify more complex anatomical structures, including the intraluminal thrombus, vascular calcifications, and branch vessels. More recently, deep learning-based models have also been applied to the segmentation of aortic dissections, enabling automatic differentiation between the true and false lumens as well as three-dimensional reconstruction of the entire aortic tree [15].

Owing to these advances, modern automated segmentation methods are now capable of accurately segmenting the entire aorta, from the ascending aorta to the iliac arteries, while substantially reducing processing time and operator-dependent variability. These developments have promoted greater standardization of quantitative vascular analysis and established the foundation for advanced applications, including patient-specific preoperative planning, computational hemodynamic simulations, radiomics, and artificial intelligence-assisted clinical decision support.

The principal automated segmentation techniques and current applications of artificial intelligence in aortic imaging are discussed in the following sections [16].

### 3.2. Clinical Utility of Segmentation

The clinical importance of medical image segmentation lies in its ability to transform complex anatomical images into quantitative, reproducible data.

In the setting of aortic disease, this capability is particularly valuable for endovascular procedure planning, where accurate anatomical characterization is essential for achieving optimal treatment outcomes. Segmentation enables precise measurements of vascular diameters, aneurysm neck length, aortic tortuosity, and the spatial orientation of branch vessels. These parameters are fundamental for endograft sizing, assessment of proximal and distal landing zones, and planning of endovascular procedures such as EVAR, FEVAR, and BEVAR.

Beyond preoperative planning, segmentation provides a more comprehensive assessment of aneurysm progression than conventional linear measurements based solely on the maximum aneurysm diameter. Volumetric quantification of the aneurysm sac and ILT allows more accurate monitoring of disease progression and post-treatment aortic remodeling.

Furthermore, the availability of accurate patient-specific three-dimensional models has paved the way for advanced computational modeling techniques. Segmented datasets can be converted into patient-specific meshes suitable for CFD simulations, finite element analysis (FEA), and other biomechanical investigations. These approaches enable quantitative assessment of hemodynamic and biomechanical parameters, including wall shear stress, pressure distribution, and flow patterns, thereby providing deeper insights into the mechanisms underlying the development and progression of aortic disease [17].

Another major advantage of segmentation is the improved reproducibility of quantitative measurements. Conventional manual measurements are inherently operator-dependent and may vary according to the reconstruction plane selected for analysis. In contrast, semi-automatic and fully automated workflows reduce inter-operator variability, improve measurement consistency, and facilitate standardized longitudinal follow-up across different institutions. Table 1 summarizes the main clinical applications of 3D aortic segmentation in AAA management, linking each application to the quantitative features that can be extracted from CTA datasets.

### 3.3. Structures That Can Be Segmented in Aortic CTA

Aortic CTA enables the segmentation of a wide range of anatomical and pathological structures; each associated with distinct clinical and research applications. Although the aortic lumen remains the most frequently segmented structure, modern segmentation techniques now allow comprehensive characterization of the entire aortic anatomy and its pathological alterations.

In aneurysmal disease, segmentation can be applied to both the aortic lumen and the outer aortic wall, allowing differentiation between the perfused lumen and the total aneurysm sac volume. This distinction is clinically relevant, as the true dimensions of an aneurysm may differ substantially from measurements based solely on the residual patent lumen. Lumen segmentation also enables extraction of the aortic centerline and calculation of key geometric parameters, including orthogonal diameters, aneurysm neck length, and vascular tortuosity, all of which are essential for endograft sizing and preoperative planning of endovascular procedures.

Within the aneurysm sac, both the ILT and vascular calcifications can be accurately segmented. Traditionally regarded as a passive filling material, ILT is now recognized as a biologically active structure involved in inflammatory processes, proteolytic activity, and aneurysmal wall remodeling [18]. Volumetric segmentation of ILT allows quantitative assessment of its burden and spatial distribution, parameters that are increasingly being investigated for their potential association with aneurysm growth and rupture risk. Similarly, segmentation of vascular calcifications enables quantification of calcification burden and assessment of its impact on arterial stiffness, the quality of proximal and distal landing zones, and the feasibility of endovascular access [19].

Segmentation is not limited to the aneurysm itself. Branch vessels, including the celiac trunk, superior mesenteric artery, renal arteries, and internal iliac (hypogastric) arteries, are routinely segmented during complex aortic procedures to accurately define their origin, course, and three-dimensional orientation. This information is essential for planning FEVAR and BEVAR as well as for the design of patient-specific endovascular devices. Likewise, segmentation of the iliac and femoral arteries enables detailed evaluation of vessel diameter, tortuosity, stenosis, and calcification burden, all of which directly influence procedural feasibility and access strategy.

Although most clinical applications of segmentation have been developed in the setting of aortic aneurysms, these techniques have also become increasingly important in the management of aortic dissection. In this context, segmentation enables separate reconstruction of the true lumen (TL), false lumen (FL), and intimal flap, providing detailed three-dimensional characterization of the dissected aorta. Quantification of TL and FL volumes, assessment of branch vessel involvement, and identification of primary entry and re-entry tears provide valuable information for therapeutic planning and prognostic stratification. Furthermore, segmentation of these structures represents a prerequisite for the generation of patient-specific computational models and the analysis of the hemodynamic behavior of the dissected aorta.

Segmentation also plays a central role in postoperative surveillance. Following EVAR, FEVAR, BEVAR, or TEVAR, both the endograft and the residual aneurysm sac can be segmented to quantitatively assess aneurysm remodeling, graft migration, branch vessel patency, and the presence of endoleaks, thereby enabling more accurate, objective, and standardized longitudinal follow-up.

More recently, interest has expanded beyond the vascular lumen to include extravascular tissues. In particular, perivascular adipose tissue (PVAT) has emerged as a promising imaging biomarker of vascular inflammation and remodeling. Indeed, PVAT-derived factors participate in all stages of pathological AAA formation, including inflammatory cell infiltration, oxidative stress onset, matrix metalloproteinase activation, and VSMC phenotype switching [20]. Quantitative characterization of PVAT using CTA may provide additional insights into the biological activity of aortic disease and currently represents one of the most rapidly evolving areas of research in quantitative vascular imaging. Among these metrics, the fat attenuation index (FAI), derived from CT attenuation values of perivascular adipose tissue, may provide a quantitative surrogate of local vascular inflammation, with higher attenuation values reflecting inflammatory remodeling of the perivascular fat compartment.

## 4. 3D Segmentation in Aortic Disease: Methods, Clinical Applications, and Artificial Intelligence

Three-dimensional aortic segmentation is a multi-step process that transforms the raw data acquired from CTA into patient-specific digital models suitable for quantitative analysis and clinical decision-making pathways [21].

Although individual workflows may vary depending on the software platform and the degree of automation available, the segmentation process generally follows a well-defined sequence of steps, commonly referred to as the segmentation pipeline. This pipeline typically includes image acquisition, dataset pre-processing, anatomical structure segmentation, three-dimensional reconstruction, extraction of quantitative biomarkers, and their subsequent integration into clinical applications.

### 4.1. General Workflow of 3D Aortic Segmentation

The segmentation process begins with the acquisition of a high-resolution contrast-enhanced CTA scan, which represents the fundamental prerequisite for accurate segmentation. CTA should be acquired during the arterial phase using thin-slice reconstructions (0.5–1.0 mm) to ensure high spatial resolution and reliable three-dimensional reconstruction. In addition, image acquisition should encompass the entire vascular territory of interest, including the proximal and distal segments intended for landing zones, branch vessels, and, when appropriate, the access vessels. This comprehensive coverage enables complete morphological assessment, appropriate device planning, and accurate postoperative surveillance.

Image quality is a critical determinant of the entire segmentation workflow, as motion artifacts, inadequate or heterogeneous contrast enhancement, and insufficient spatial resolution may compromise subsequent processing steps and reduce segmentation accuracy.

**Figure 2:**
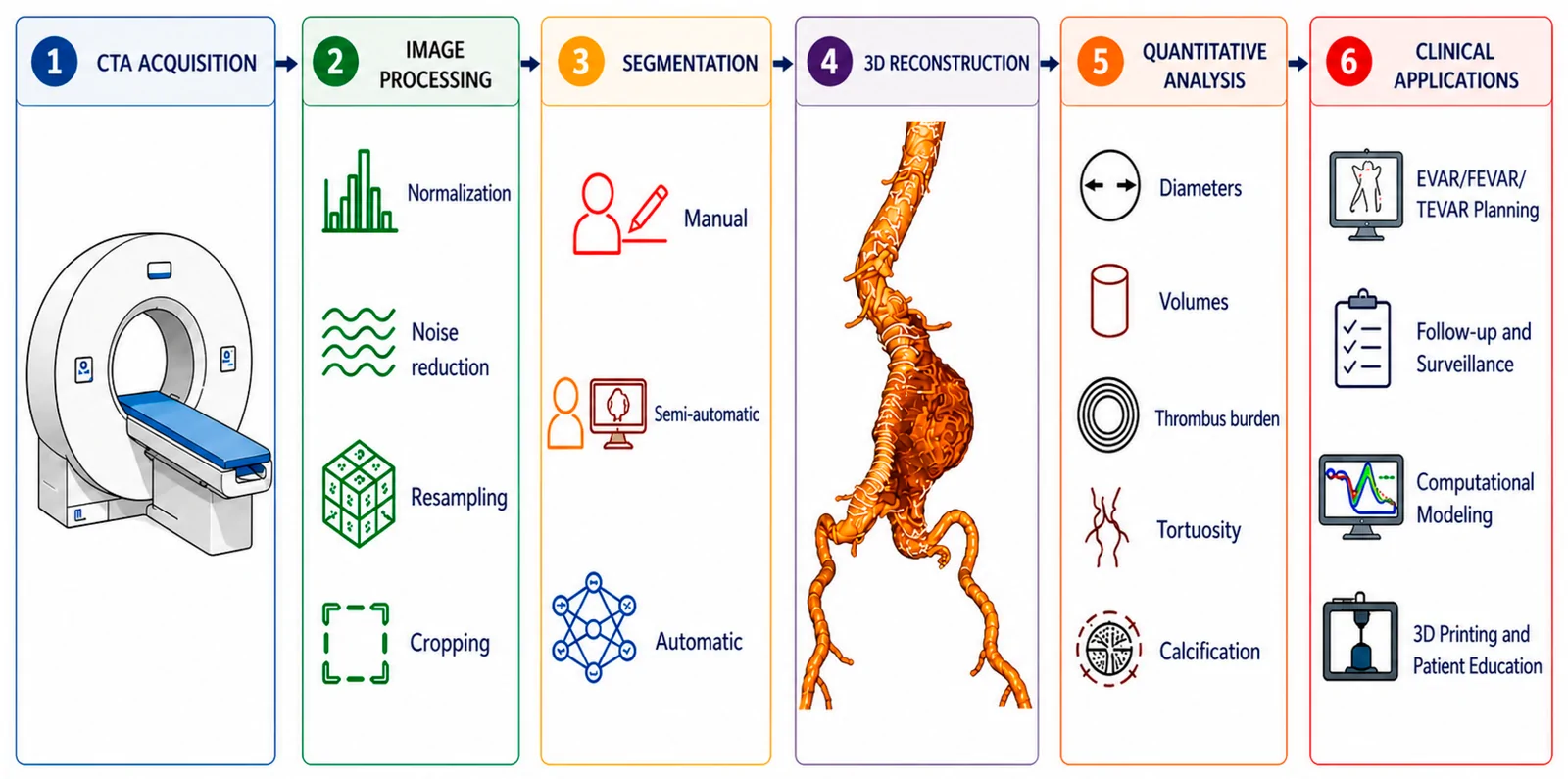
General workflow of CTA-based aortic segmentation from image acquisition and preprocessing three-dimensional reconstruction, quantitative analysis, and clinical review. (CTA: computed tomography angiography; EVAR: endovascular aneurysm repair; FEVAR: fenestrated endovascular aneurysm repair; TEVAR: thoracic endovascular aneurysm repair). Volume-rendered aortic illustration in panel 4 is not derived from patient data and it is used solely for educational purpose.

Before segmentation, the CTA dataset undergoes a series of preprocessing procedures aimed at optimizing and standardizing the input data [21,22]. This stage is particularly important because image characteristics may vary according to the CT scanner, acquisition parameters, slice thickness, and contrast administration protocol. The primary objective is to minimize inter-scan variability and establish optimal conditions for subsequent image analysis. Figure 2 summarizes the general workflow steps commonly used.

The most commonly performed preprocessing operations include:

- Intensity normalization, which standardizes voxel intensity values across different acquisitions, thereby reducing technical variability related to imaging protocols.
- Noise reduction, which suppresses image noise and artifacts while preserving relevant anatomical boundaries, ultimately improving segmentation accuracy.
- Isotropic resampling, which reconstructs the dataset onto a uniform three-dimensional grid in which all voxels have identical dimensions. This process improves the geometric representation of vascular structures and enhances the reliability of three-dimensional reconstructions and quantitative measurements, including vessel length, diameter, volume, and tortuosity. Furthermore, isotropic resampling is frequently a prerequisite for deep learning algorithms, which require spatially homogeneous input data to achieve optimal performance [23].
- Region-of-interest (ROI) cropping, which restricts the analysis to the relevant anatomical region, such as the thoracic aorta, abdominal aorta, or the entire aortoiliac axis. By excluding irrelevant anatomical structures, ROI cropping reduces computational burden and enables segmentation algorithms to focus on the vascular structures of interest.

**Figure 3:**
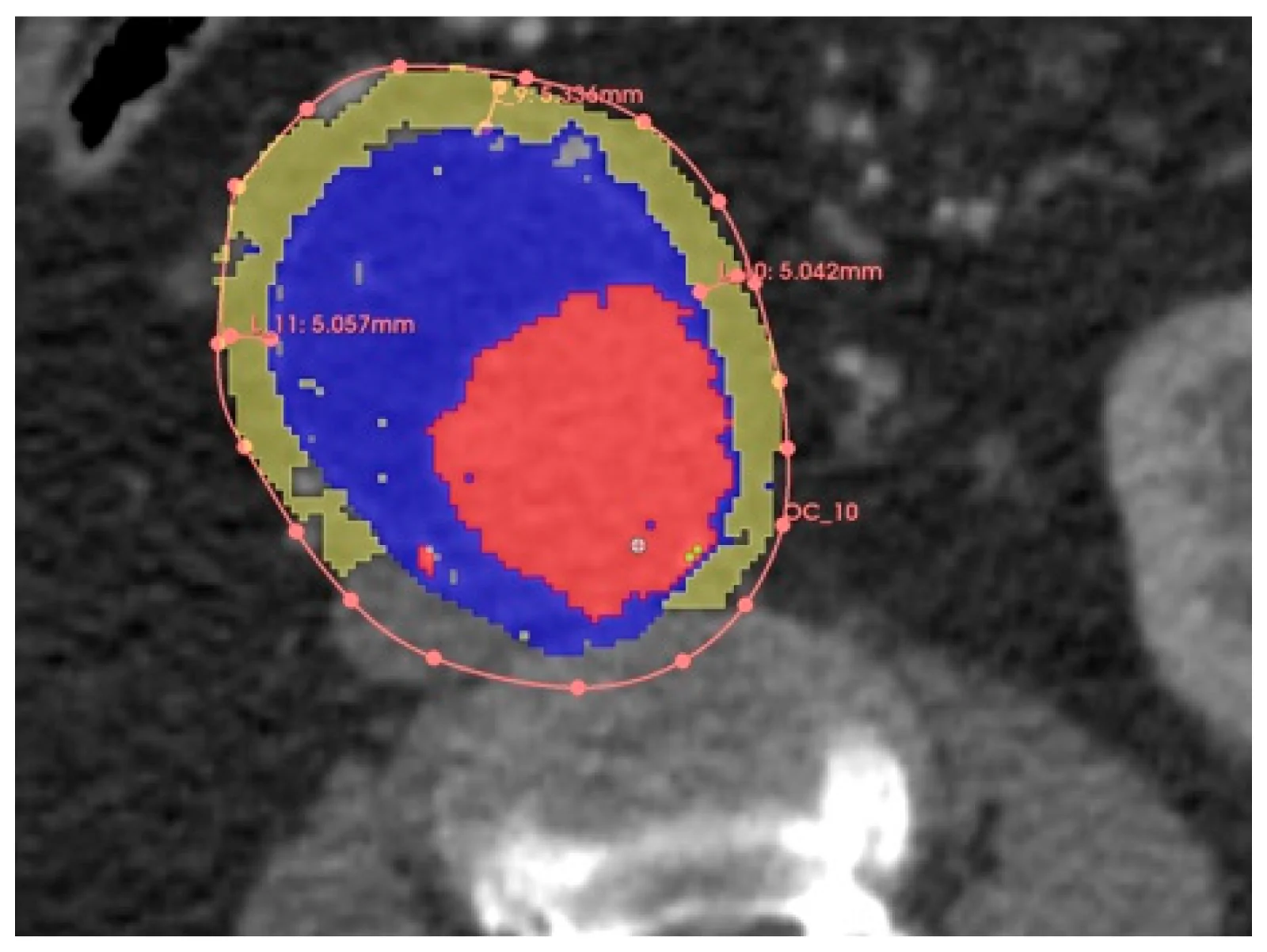
De-identified axial arterial-phase CTA image of an infrarenal abdominal aortic aneurysm, manual slice-by-slice segmentation. The red region represents the contrast-filled aortic lumen, the blue region represents intraluminal thrombus, and the yellow-green peripheral region represents the manually delineated perivascular adipose tissue (PVAT). The colored red contour and control points indicate the manually placed boundaries used for segmentation and diameter assessment; the numerical labels report local distances in millimeters for PVAT localization. The annotations and measurements were manually created by the authors in 3D Slicer (version 5.8.1; open-source software). The CTA image was acquired during routine care at Azienda Ospedaliero-Universitaria Senese, Siena, Italy, and was annotated by the authors. The displayed image was de-identified and is presented solely as an educational illustration.

The central stage of the workflow is the segmentation process itself, during which the anatomical structures of interest are identified and separated from the surrounding tissues. An example of manual slice-by-slice segmentation on an axial CTA image is shown in Figure 3. Depending on the methodology employed, segmentation may be performed manually through slice-by-slice delineation by the operator or by means of semi-automatic or fully automated approaches.

The resulting segmentation masks are subsequently converted into three-dimensional models using surface reconstruction algorithms. During this stage, digital surfaces and polygonal meshes can be generated and exported to dedicated software platforms for clinical planning, computational modeling, or three-dimensional printing. Simultaneously, the aortic centerline and orthogonal cross-sectional planes along the vascular course are typically extracted, providing the basis for accurate morphometric analysis.

Three-dimensional reconstruction serves as the foundation for the extraction of quantitative imaging biomarkers, including orthogonal vessel diameters, cross-sectional areas, aneurysm volume, intraluminal thrombus burden, vascular tortuosity, aneurysm neck length, and the geometric characteristics of branch vessels.

Finally, these quantitative data are integrated into clinical decision-making, supporting the planning of EVAR/TEVAR and F/BEVAR procedures, postoperative surveillance, and advanced applications such as CFD, FEA, and patient-specific three-dimensional printing.

### 4.2. Segmentation Methods

Over the past two decades, aortic segmentation techniques have undergone a remarkable evolution, progressing from entirely manual approaches to highly automated workflows driven by AI. This evolution has been motivated by the need to reduce processing time, improve measurement reproducibility, and enable the analysis of increasingly complex imaging datasets. Currently, segmentation methods can be broadly classified into three main categories: manual, semi-automatic, and fully automatic approaches.

#### 4.2.1. Manual Approaches

Manual segmentation consists of the direct delineation of anatomical structures by the operator on each CTA slice. The user manually outlines the vascular lumen, aneurysm wall, ILT, or other structures of interest throughout the entire imaging volume, generating a three-dimensional segmentation mask by combining the slice-by-slice annotations. Although this approach requires considerable expertise and processing times that are often incompatible with routine clinical practice, it remains the methodological reference standard (gold standard) for the generation of annotated datasets used to develop and validate machine learning and deep learning algorithms [22].

Manual segmentation provides a high degree of anatomical control and remains particularly valuable in cases involving complex vascular anatomy, extensive aortic dissections, endoleaks, metallic artifacts, or other challenging conditions in which automated algorithms may yield suboptimal performance.

Among the software platforms most commonly used for manual medical image segmentation are ITK-SNAP (Cognition and Communication Laboratory, University of Pennsylvania, Philadelphia, PA, USA), 3D Slicer (Brigham and Women’s Hospital, Boston, MA, USA), and Materialise Mimics (Materialise NV, Leuven, Belgium), together with several commercial workstations and PACS platforms equipped with dedicated vascular post-processing tools [24]. In particular, ITK-SNAP integrates both manual and semi-automatic segmentation tools based on active contour algorithms, enabling efficient delineation of three-dimensional anatomical structures [25]. 3D Slicer, an open-source platform widely adopted in both research and clinical practice, provides advanced tools for multiplanar visualization, segmentation, three-dimensional reconstruction, and quantitative analysis of medical images [26]. Materialise Mimics is one of the leading commercial platforms for medical image segmentation and is extensively used for preoperative planning, patient-specific modeling, and three-dimensional printing.

These software platforms allow simultaneous visualization of axial, sagittal, and coronal reconstructions while enabling manual refinement of segmentation masks throughout the entire segmentation process. For several of the aforementioned platforms, semi-automatic analysis tools are available.

The principal limitations of manual segmentation include its time-consuming nature, intra- and inter-observer variability, and the difficulty of achieving standardized quantitative measurements across different institutions [22,25].

#### 4.2.2. Semi-Automatic Approaches

To overcome the limitations of fully manual segmentation, numerous semi-automatic methods have been developed, in which the operator provides an initial input while the software automatically performs part of the segmentation process [22,23].

**Figure 4:**
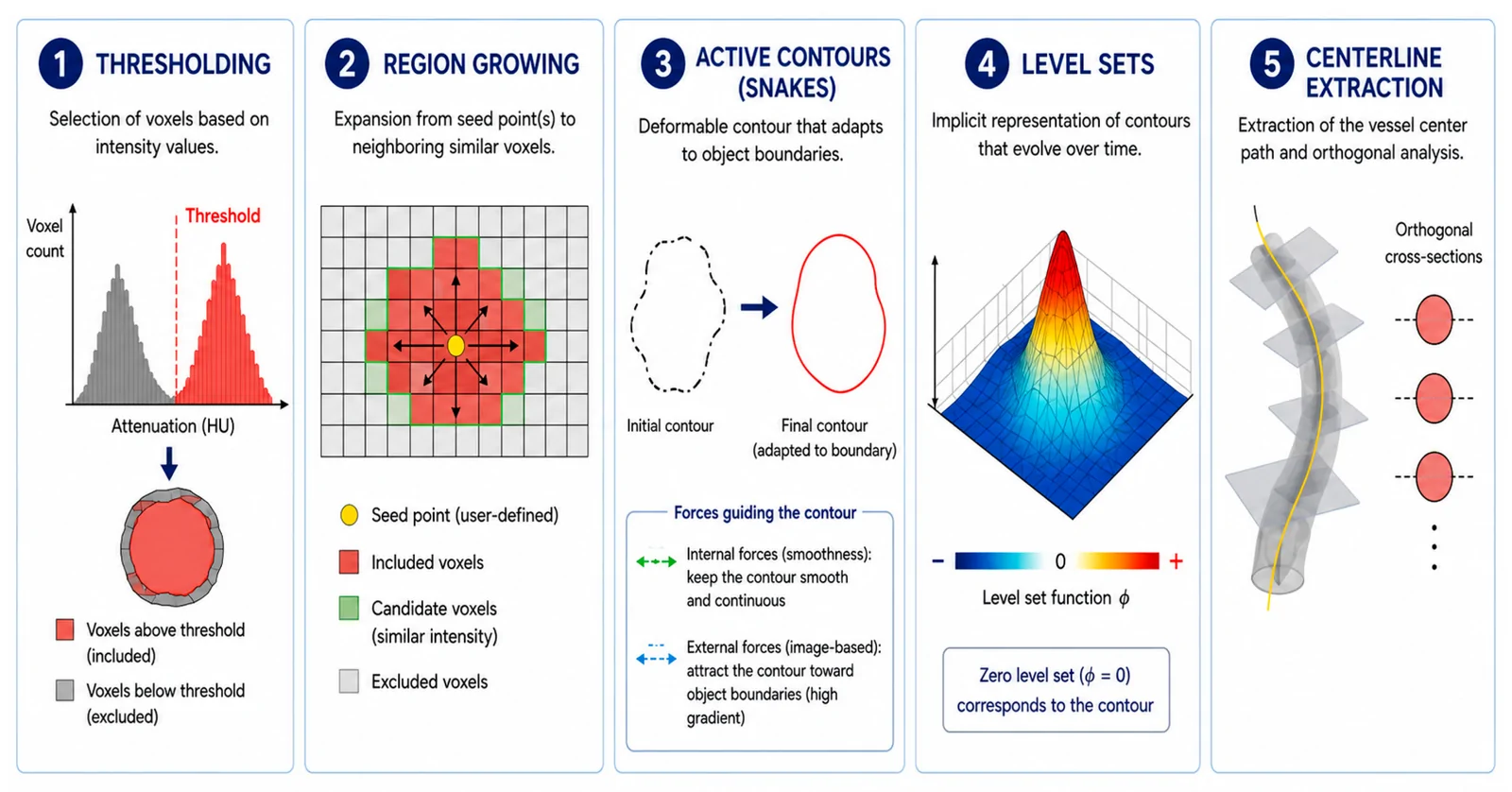
Overview of the principal semi-automatic segmentation methods used for CTA-based aortic segmentation, including thresholding, region growing, active contours, level sets, and centerline extraction.

The most widely used semi-automatic techniques include thresholding, region growing, active contour models, level-set methods, and centerline extraction algorithms (Figure 4).

Thresholding is one of the simplest segmentation approaches and is based on the classification of voxels according to their radiological attenuation values. One or more attenuation thresholds, expressed in HU, are defined, and all voxels falling within the selected range are assigned to the anatomical structure of interest. In CTA, this method is particularly effective for the preliminary identification of the contrast-enhanced vascular lumen but may be less accurate in the presence of extensive calcifications, intraluminal thrombus, or heterogeneous contrast enhancement [27]. Region growing extends the thresholding approach by using one or more operator-defined seed points. Starting from these initial locations, the algorithm iteratively expands the segmented region by incorporating neighboring voxels that satisfy predefined similarity criteria, typically based on attenuation values, spatial connectivity, and anatomical continuity. Compared with simple threshold-based segmentation, region growing is better suited to the complex morphology of vascular structures [6]. Active contour models, commonly referred to as snakes, employ deformable curves initialized in close proximity to the target structure. During an iterative optimization process, the contour evolves under the influence of internal forces, which preserve its smoothness and geometric continuity, and external image-derived forces, such as intensity gradients and anatomical boundaries. The objective is to achieve an accurate fit between the evolving contour and the true borders of the anatomical structure [9]. Level-set methods represent a mathematical extension of active contour models. In this approach, the contour is not explicitly represented but is instead defined as the zero level of a three-dimensional scalar function (φ). The evolution of the level-set function allows robust handling of complex topological changes, including merging and splitting of segmented regions, making these algorithms particularly suitable for the segmentation of anatomically irregular structures [21].

A central component of vascular image analysis is represented by centerline extraction algorithms, which automatically identify the geometric center of the vessel along its entire course. Once the centerline has been generated, the software can reconstruct cross-sectional images orthogonal to the vessel axis and perform standardized measurements of vessel diameter, cross-sectional area, length, angulation, and tortuosity. Consequently, centerline extraction constitutes a fundamental component of most software platforms used for planning EVAR, FEVAR, BEVAR, and TEVAR procedures.

Dedicated planning platforms such as 3mensio Vascular (Pie Medical Imaging, Maastricht, The Netherlands), EndoSize (Therenva, Rennes, France), and TeraRecon Aquarius iNtuition (TeraRecon Inc., Durham, NC, USA), as well as the aforementioned manual analysis software platforms, incorporate tools for automated centerline extraction and semi-automatic segmentation of the aortic lumen and its branch vessels.

Semi-automatic approaches currently represent the most widely adopted compromise in routine clinical practice, as they substantially reduce processing time while maintaining direct operator supervision. Nevertheless, their performance may deteriorate in the presence of extensive intraluminal thrombus, severe vascular calcifications, complex aortic dissections, or low-contrast imaging, often requiring manual refinement of the segmentation masks [8,9].

#### 4.2.3. Automatic Workflows

Fully automatic workflows represent the most recent evolution in aortic image segmentation. In these approaches, the entire segmentation process is performed without direct operator intervention: the software receives the CTA dataset as input and automatically generates one or more segmentation masks of the anatomical structures of interest [23,28]. Over the past decade, advances in machine learning and, in particular, deep learning have markedly improved the performance of these systems, enabling fully automated segmentation of the aorta and its major branch vessels with increasingly high levels of accuracy.

The introduction of CNN architectures, followed by dedicated biomedical segmentation models such as U-Net, has represented a major milestone in the development of fully automated segmentation systems. Unlike conventional image-processing methods, these architectures are capable of learning anatomical features directly from imaging data without requiring manually engineered (hand-crafted) features.

In contrast to manual and semi-automatic approaches, in which the operator explicitly or implicitly defines the region of interest, fully automated workflows learn the anatomical characteristics of the target structures from annotated datasets during the training phase. Once training has been completed, the model can automatically identify and segment the aortic lumen, vessel wall, intraluminal thrombus, and other relevant anatomical structures within the CTA examination [8,29].

Despite the remarkable performance achieved by modern deep learning algorithms, human supervision remains essential, particularly in the presence of complex anatomy, extensive aortic dissections, imaging artifacts, inadequate contrast enhancement, or previous surgical or endovascular interventions. Consequently, in current clinical practice, fully automated algorithms are most commonly used as pre-segmentation tools, with the resulting segmentation masks subsequently reviewed and, when necessary, manually refined by the operator [16].

The latest advances in this field extend beyond automated segmentation itself and focus on the development of end-to-end workflows, in which artificial intelligence integrates the major steps of vascular image analysis into a single automated pipeline. Within these systems, segmentation constitutes the initial stage of a comprehensive workflow that also includes three-dimensional reconstruction of the aorta, automatic centerline extraction, computation of morphological and geometric biomarkers, and, in selected applications, generation of patient-specific models for computational hemodynamic simulations [9,30].

At present, these end-to-end workflows are primarily used in research settings but are being progressively incorporated into next-generation platforms for quantitative cardiovascular image analysis. Their ultimate goal is to automate the entire process—from raw CTA data to the generation of quantitative imaging biomarkers and patient-specific three-dimensional models—thereby minimizing operator dependence while improving the reproducibility and standardization of image analysis.

### 4.3. Artificial Intelligence in Aortic CTA Segmentation

#### 4.3.1. Evolution of AI in Medical Image Segmentation

The fully automated workflows described in the previous section provide the framework within which AI has progressively transformed medical image segmentation. Unlike traditional segmentation methods, in which predefined rules are applied using techniques such as thresholding, region growing, active contours, or level-set algorithms, AI-based models are trained on large datasets of CTA examinations that have been previously annotated by expert operators, serving as the ground truth. Expert annotations provide the reference standard for model training and evaluation. However, expert assessment is not free from variability: differences between observers have been documented in CTA interpretation and anatomical measurements in patients with suspected ruptured AAA [5].

During the training phase, the algorithm progressively learns to recognize the anatomical characteristics associated with the different structures of interest, including the aortic lumen, vessel wall, intraluminal thrombus, vascular calcifications, and branch vessels, without these features being explicitly programmed by the operator. Rather than relying on predefined rules, the model automatically learns hierarchical image representations directly from the training data, enabling the recognition of increasingly complex anatomical patterns and improving its ability to generalize to previously unseen CTA examinations [25].

Once training has been completed, the model can be applied to new CTA datasets to automatically perform image segmentation, generate patient-specific three-dimensional models, and extract quantitative imaging biomarkers with high accuracy and reproducibility. Modern AI-based algorithms are capable of segmenting not only the aortic lumen but also the vessel wall, intraluminal thrombus, vascular calcifications, and major branch vessels, thereby supporting the development of fully automated pipelines for preoperative planning and postoperative surveillance of aortic diseases.

The transition from rule-based to data-driven approaches has represented one of the most significant advances in the evolution of aortic image segmentation. By learning directly from imaging data rather than relying on manually engineered rules, AI algorithms have substantially improved the automation, robustness, and scalability of segmentation workflows, paving the way for increasingly integrated quantitative cardiovascular imaging platforms.

An early AAA-specific application was DeepAAA, which combined a modified 3D U-Net with ellipse fitting for abdominal aortic segmentation and aneurysm detection on CT images [31].

#### 4.3.2. From Artificial Intelligence to Deep Learning

**Figure 5:**
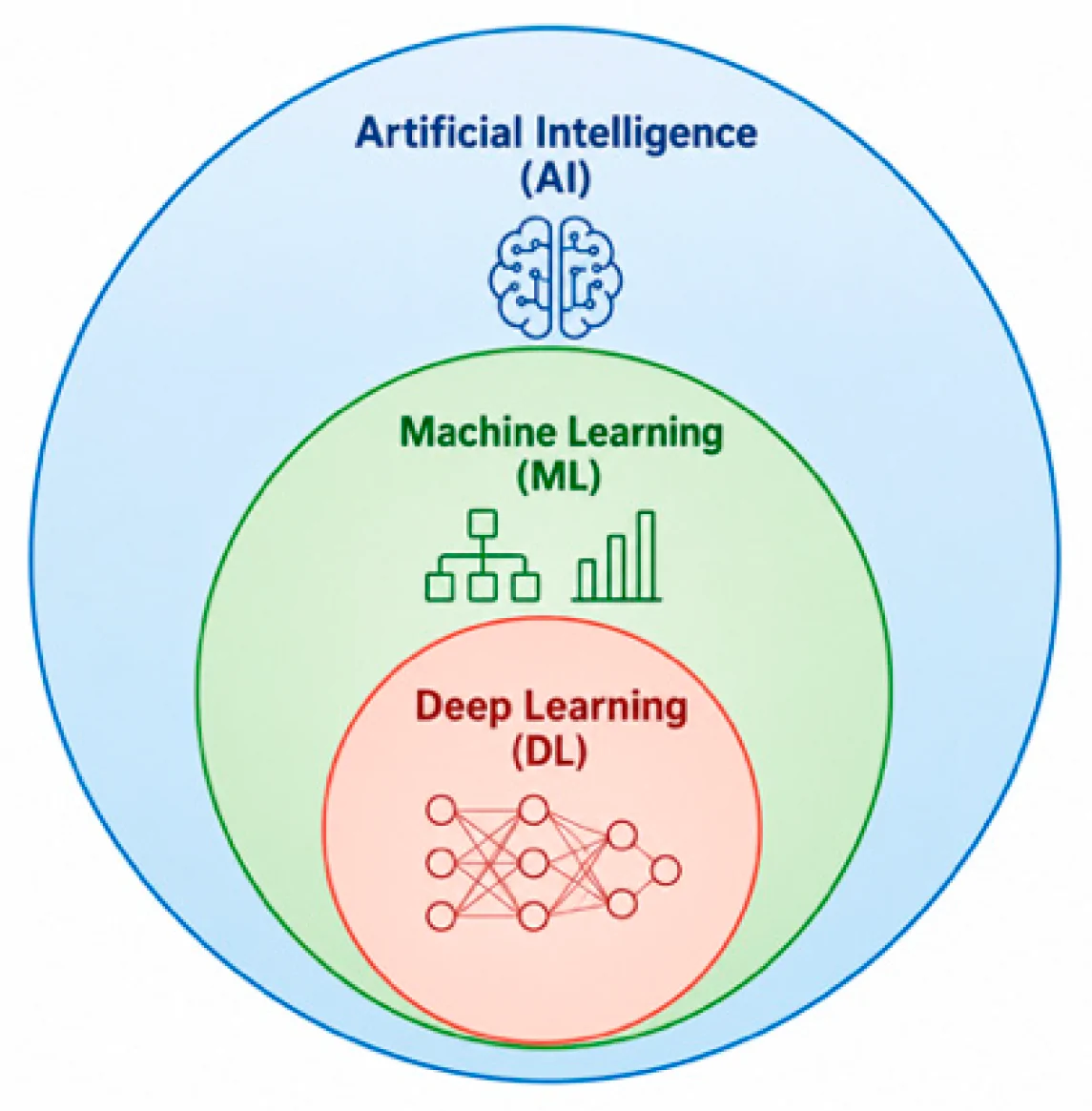
Hierarchical relationship between Artificial Intelligence (AI), Machine Learning (ML), and Deep Learning (DL).

To understand the role of artificial intelligence in automated aortic segmentation, it is important to distinguish between the concepts of AI, Machine Learning (ML), and DL, which represent hierarchical levels within the same technological framework (Figure 5).

AI encompasses computer systems designed to perform tasks that would typically require human cognitive abilities, including image recognition, classification, learning, and clinical decision support. Within AI, ML refers to a subset of algorithms capable of learning from data without being explicitly programmed through predefined rules. In aortic image segmentation, ML models are trained using CTA datasets previously annotated by expert operators, enabling them to recognize the anatomical characteristics of the aorta and surrounding vascular structures.

DL represents the most advanced branch of Machine Learning and is based on artificial neural networks (ANNs) composed of multiple computational layers capable of automatically learning increasingly complex hierarchical representations of imaging data. Unlike traditional image analysis methods, deep learning does not require manually engineered (hand-crafted) features; instead, relevant anatomical features are learned directly from the training data, resulting in substantial improvements in medical image segmentation performance.

During the training phase, the model is exposed to a large collection of CTA examinations that have been manually annotated by expert operators, providing the ground truth for supervised learning. Through an iterative optimization process, the predicted segmentation is continuously compared with the reference annotation, and the network progressively updates its internal parameters to minimize the discrepancy between the two. As training proceeds, the model learns to accurately recognize the anatomical characteristics of the aorta and surrounding structures, allowing it to generalize to previously unseen CTA examinations. Among the various deep learning architectures developed for medical image analysis, CNNs have become the reference standard for automated image segmentation. By applying convolutional filters, CNNs progressively learn image features of increasing complexity, ranging from low-level characteristics such as edges and textures to high-level anatomical structures, making them particularly effective for the automatic segmentation of medical images [23,25].

Among CNN-based architectures, the U-Net family, specifically developed for biomedical image segmentation, remains one of the most widely adopted approaches. Its characteristic U-shaped architecture consists of a contracting path (encoder) and an expanding path (decoder) connected through skip connections, which combine local spatial information with high-level semantic features, thereby improving the delineation of anatomical boundaries [23]. Over the years, numerous variants, including U-Net++, Attention U-Net, and ResU-Net, have been proposed to further enhance segmentation accuracy. More recently, considerable attention has been directed toward nnU-Net, a self-configuring deep learning framework that automatically adapts the network architecture, preprocessing strategy, and training parameters to the characteristics of the input dataset. By minimizing the need for manual optimization, nnU-Net has achieved state-of-the-art performance across a broad range of biomedical image segmentation tasks and has become an important benchmark for the development of new segmentation algorithms.

In parallel, foundation models are emerging as a promising new paradigm in medical image analysis. These large-scale models are pre-trained on extensive and heterogeneous imaging datasets and can subsequently be adapted to specific clinical applications through transfer learning. Although their application to cardiovascular imaging is still in its early stages, foundation models are expected to play a key role in the future development of automated aortic segmentation and quantitative cardiovascular imaging.

#### 4.3.3. Advantages of AI-Based Segmentation

The introduction of AI into automated aortic segmentation has profoundly transformed the CTA image analysis workflow, offering advantages that extend well beyond the automation of anatomical delineation. The main benefits include reduced processing time, improved standardization and reproducibility of measurements, the ability to analyze large imaging datasets, and the automated extraction of quantitative imaging biomarkers for treatment planning and postoperative surveillance.

One of the most evident advantages is the substantial reduction in processing time. Whereas manual segmentation often requires tens of minutes and is highly dependent on operator expertise, modern deep learning algorithms can generate complete segmentations within a fraction of that time, making automated image analysis increasingly compatible with routine clinical workflows [9,16].

Wang et al. reported a pooled Dice similarity coefficient of 0.96 (95% CI: 0.95–0.96) across 16 studies of aortic CT segmentation. This estimate reflects studies with heterogeneous anatomical coverage and should not be interpreted as an infrarenal AAA-specific benchmark. Although one study was explicitly classified as addressing the abdominal aorta, the anatomical descriptions provided did not permit a reliable count of studies involving the infrarenal segment. No pooled estimate specific to infrarenal aortic or AAA segmentation was reported [21]. The pooled Dice similarity coefficient represents the combined estimate, across multiple studies, of the spatial overlap between automated and expert-derived segmentations. High Dice scores indicate substantial volumetric overlap but do not necessarily imply accurate local boundaries or clinically reliable measurements. In large structures such as the aorta, a high Dice score may coexist with local segmentation discrepancies that affect neck diameter, neck length, or landing-zone assessment.

Results from thoracic or whole-aorta studies cannot be directly extrapolated to infrarenal AAA. Thrombus, mural calcification, and iliac tortuosity create additional segmentation challenges that require validation in the intended anatomical region and patient population.

Another important advantage is the improved standardization of anatomical measurements. Automated segmentation substantially reduces the intra- and inter-observer variability inherent to manual analysis, thereby improving the reproducibility of morphometric measurements and enabling more reliable comparisons between examinations acquired at different time points and across different institutions [22,32].

This feature is particularly valuable in multicenter studies and in the longitudinal follow-up of patients undergoing endovascular aortic repair.

AI-based segmentation also enables the efficient processing of large patient cohorts, allowing thousands of CTA examinations to be analyzed within timeframes compatible with large-scale observational studies and the development of predictive models based on quantitative imaging data. At the same time, automated segmentation facilitates the systematic extraction of morphological and geometric imaging biomarkers, including orthogonal vessel diameters, cross-sectional areas, aneurysm volume, intraluminal thrombus burden, vascular tortuosity, and branch vessel morphology, thereby expanding the potential of quantitative cardiovascular imaging.

A representative example is provided by the study of Weiss et al., who developed an nnU-Net-based framework for the automated segmentation of abdominal aortic aneurysms in both the preoperative and post-endovascular settings [33]. Their model achieved Dice similarity coefficients exceeding 0.96 for both the aneurysm sac and the aortic lumen, demonstrating excellent agreement with manual segmentation while reducing image analysis time by more than 50%, thereby highlighting the considerable clinical potential of AI-based segmentation tools.

Finally, automated segmentation has evolved from an isolated image-processing task into the initial stage of fully integrated imaging workflows [14,15,17].

Integrated platforms such as AortaAnalyzer, an open-access research platform developed by von Deylen et al. [34] or TotalSegmentator by Wasserthal et al. [35], illustrate how automated segmentation, quantitative morphometric analysis, and computational modeling can be incorporated into a single end-to-end workflow, reducing operational complexity while promoting faster, more standardized, and highly reproducible image analysis.

Table 2 summarizes the main technical architectures and clinical-operational models currently available for AI-based aortic CTA segmentation.

## 5. Limitations and Barriers

Despite the remarkable advances achieved in recent years, the clinical implementation of AI for aortic image segmentation continues to face important technical, methodological, and regulatory challenges that limit its widespread adoption in routine clinical practice.

One of the major limitations relates to the dependence of AI models on the quality of the training data. The performance of a segmentation algorithm is strongly influenced by the quality, size, and representativeness of the datasets used during training. Models developed using data from a single institution or acquired with homogeneous imaging protocols may exhibit limited generalizability when applied to different patient populations or imaging environments. Closely related to this issue is the phenomenon of domain shift, which occurs when a trained model is applied to CTA examinations acquired using different CT scanners, acquisition protocols, contrast enhancement strategies, or patient populations than those represented in the training dataset. Under these circumstances, segmentation performance may deteriorate substantially, highlighting the need for additional adaptation strategies and rigorous external validation [24].

Public resources already support aortic segmentation research, including the multicenter AVT dataset [36] and the MICCAI SEG.A. 2023 [37]. However, performance on these benchmarks does not establish reliability for all AAA-related targets or post-EVAR anatomy, which require dedicated validation.

Another important limitation of deep learning models is their limited interpretability, commonly referred to as the “black-box” problem. Although these algorithms can achieve excellent segmentation accuracy, the decision-making process leading to the final prediction often remains opaque. Consequently, it is difficult to determine which image features influenced the identification of anatomical structures or contributed to segmentation errors. This limitation is particularly relevant in clinical practice, where understanding the rationale underlying algorithmic decisions is essential to ensure trust, patient safety, and clinical accountability.

To address this challenge, the concept of Explainable Artificial Intelligence (XAI) has emerged, encompassing a range of methodologies designed to improve the interpretability of deep learning models [25].

Techniques such as saliency maps, Gradient-weighted Class Activation Mapping (Grad-CAM), and heatmaps can highlight the image regions that most strongly influenced the segmentation process, thereby providing clinicians with valuable insights into model behavior. Although these approaches do not completely resolve the black-box problem, they represent a promising strategy for improving the transparency, trustworthiness, and clinical acceptance of AI-based systems.

**Aortic-specific limitations.** Aortic CTA segmentation presents failure modalities that are not fully captured by generic discussions of medical AI. At the infrarenal neck, calcium blooming may obscure or displace the apparent lumen boundary, producing errors of only a few millimeters that may nevertheless affect neck-diameter measurements, landing-zone assessment, and endograft sizing. In post-EVAR surveillance, metallic components may generate streak and blooming artefacts that degrade segmentation around the endograft and adjacent lumen. Metal artefacts from a deployed endograft may obscure the lumen and aneurysm sac boundaries on post-EVAR CTA, reducing segmentation accuracy and requiring careful expert review.

Endoleak assessment also requires appropriate acquisition timing, including delayed or multiphasic CTA in many clinical workflows; therefore, a model trained exclusively on arterial-phase images cannot be assumed to provide validated automated endoleak detection. Small structures, including calcified plaques and accessory renal arteries, occupy relatively few voxels, making their Dice scores more unstable and less informative than those of the contrast-filled lumen. Boundary distances and clinically relevant measurement errors should therefore complement volumetric overlap metrics.

Cohort composition is another important limitation. AAA datasets are frequently composed predominantly of male patients. Because women may have smaller aortic dimensions and different device-related anatomical margins, a model trained on a male-dominated distribution may perform less reliably in female patients. Future studies should report sex-specific cohort composition, evaluate performance separately by sex, and include external validation in populations with different demographic and anatomical distributions.

Regulatory and clinical validation issues also remain significant barriers to implementation. Most AI algorithms reported in the literature have been developed and evaluated in single-center studies, whereas evidence from prospective, multicenter validation studies involving heterogeneous patient populations remains limited. Before these technologies can be adopted on a large scale, their robustness, reproducibility, and safety must be demonstrated across diverse clinical settings. Furthermore, AI-based segmentation software must comply with the regulatory requirements governing medical devices before being integrated into routine clinical practice. Regulatory requirements for AI-enabled medical software are risk- and intended-purpose-based. In Europe, classification is governed by the EU MDR and AI Act [38], whereas in the United States FDA review may include a predetermined change-control plan for future software updates (FDA, 2025) [39].

Based on the evidence currently available, AI should therefore be regarded as a clinical decision-support tool rather than a replacement for expert clinical judgment. At present, the combination of automated algorithms with specialist supervision represents the safest and most reliable approach to ensure the accuracy, reproducibility, and appropriate clinical interpretation of results generated by automated aortic segmentation.

## 6. Future Directions

Automated pipelines combining aortic segmentation and geometric analysis illustrate the potential of AI to support endovascular planning [40].

While the current phase of research has established the ability of algorithms to match human precision in delineating anatomical contours, with processing times reduced from hours of manual work to seconds per volume, future directions aim to create a digital ecosystem in which radiological imaging ceases to be a static snapshot and becomes a predictive and personalized analytical engine [7].

### 6.1. “Human-in-the-Loop” Models and the New Clinical Oversight

In the short term, the most realistic model is represented by human-in-the-loop systems, in which artificial intelligence automatically performs presegmentation, centerline extraction, and key morphometric measurements, while the physician verifies the result, corrects any errors, and retains responsibility for the final decision. This approach combines the speed and reproducibility of automation with the practitioner’s ability to recognize complex anatomies, artifacts, and situations not represented in the training datasets. The general evidence from medical imaging suggests that human–AI collaboration can reduce the workload without compromising diagnostic performance [40].

This approach addresses three fundamental needs:

1. Accountability and Transparency: It ensures that clinical judgment remains the final decision-making authority, which is essential for medical–legal accountability [41].
2. Reliability in Rare Cases: Human intervention allows for the management of extreme anatomical variability, such as distinguishing between true and false lumens in type B aortic dissections, where voxel intensities can be nearly identical, rendering “grow from seeds” or pure automation insufficient [15,40].
3. Continuous Learning: Every manual correction made on platforms such as 3D Slicer [24] or ITK-SNAP can be fed back into the system to further refine the learning models through semi-supervised techniques, overcoming the chronic shortage of high-quality labeled data [9].

### 6.2. Digital Twins: The Dynamic Patient Replica

One of the most promising developments is digital twins, dynamic, patient-specific digital replicas of the aorta created by integrating geometry derived from CTA with clinical, hemodynamic, biomechanical, and procedural data. Unlike a static 3D reconstruction, the digital twin could simulate the interaction between the aortic wall and the endoprosthesis, predicting the device’s conformation after deployment, changes to the landing zones, and the risk of migration, bird-beak, branch occlusion, or endoleak [33].

Looking ahead, surgeons could virtually compare different devices, diameters, deployment levels, and procedural strategies prior to surgery. In the future, AI will automatically generate these digital twins from CTA, enabling the transition to “in silico medicine” [15,17]. In this scenario, endovascular treatment can be tested virtually: the surgeon can simulate the insertion of different guidewires or the deployment of various stent-graft models, predicting their mechanical behavior and interaction with the vascular wall even before entering the operating room [9].

Preliminary studies have already explored physics-informed models for predicting type IA endoleaks, but these results should be considered proof-of-concept and require validation on larger and more heterogeneous cohorts.

### 6.3. Post-EVAR Predictive Simulation and Dynamic Monitoring

Automatic segmentation will also serve as the starting point for post-EVAR predictive simulations. The integration of Computational Fluid Dynamics and Finite Element Analysis with longitudinal anatomical models could make it possible to estimate not only the immediate technical outcome, but also the subsequent remodeling of the aneurysmal sac, the persistence of pressurization, the occurrence of endoleaks, and the probability of reoperation. Multimodal models that combine preoperative imaging, device characteristics, clinical data, and follow-up results are already the subject of multicenter studies aimed at predicting the volumetric reduction of the aneurysm sac after EVAR [15].

In a physics-informed surrogate-model study, AI predicted aortic pressure and velocity fields up to 4000 times faster than conventional CFD solvers, with an estimated computation time of approximately 0.075 s per case [8]. This speed would allow the surgeon to receive immediate hemodynamic feedback during EVAR planning and in the subsequent postoperative period, identifying:

- Wall shear stress (WSS) distribution: An invisible but crucial biomarker directly correlated with the risk of post-procedural complications such as endoleaks, stent migration, or aneurysm sac expansion
- Pressure gradients: In aortic dissections, AI could predict whether the pressure in the false lumen will lead to compression of the true lumen or to organ ischemia.
- Endoleak Risk: Proactive identification of areas with turbulent flow or stagnation that could compromise the prosthesis’s seal [17].

### 6.4. Radiomics and Morphomics: Beyond Maximum Diameter

For decades, the clinical diagnosis of aneurysms has been based on maximum diameter (e.g., the 5–5.5 cm threshold). Future perspectives for AI aim to move beyond this reductionist view through automated radiomics and morphomics. These algorithms extract thousands of quantitative parameters from CTA—such as iliac tortuosity, neck angle, and ILT composition—that are imperceptible to the human eye. Integrating these “digital signatures” with demographic data will enable the definition of personalized reference diameters: since aortic diameter varies physiologically with age, sex, and body surface area, AI will be able to alert clinicians to pathological dilation even if it has not yet reached standard thresholds. Furthermore, automated analysis of thrombi and calcifications will enable the stratification of the risk of distal embolization during endovascular procedures.

### 6.5. Integration into the Clinical Workflow and Interoperability

The clinical value of these technologies will depend on their interoperability. Future platforms will need to integrate directly with PACS, vascular workstations, and electronic health records, importing DICOM data and generating masks, centerlines, measurements, and structured reports without interrupting the normal workflow. Compatibility with commercial software and open-source platforms, along with the adoption of shared formats and standardized validation procedures, will be essential for facilitating multicenter studies, model updates, and their transferability across different hospitals. In this scenario, segmentation will no longer be an isolated step but the core of a digital ecosystem capable of supporting personalized planning, device selection or customization, and follow-up tailored to individual risk.

## 7. Conclusions

CTA remains the primary imaging modality for patients undergoing evaluation for EVAR, while AI-assisted three-dimensional segmentation can transform CTA from descriptive imaging into a source of quantitative and patient-specific information. The isolation of different anatomical structures and the extraction of data allow for automated extraction of diameters, lengths, and aneurysm neck characteristics, with measurement accuracy requiring dedicated validation against expert reference measurements. Furthermore, in follow-up, volumetric analysis can provide a more comprehensive assessment of aneurysmal remodeling than measuring the maximum diameter alone. Deep learning algorithms have demonstrated high performance in automatic segmentation and a significant reduction in processing time. The most realistic near-term clinical model is human-in-the-loop AI-assisted segmentation, in which architectures such as U-Net and nnU-Net generate preliminary masks that are reviewed and validated by vascular imaging specialists. At the same time foundation models are developing, large-scale pre-trained models on vast amounts of data adaptable to specific applications through transfer learning. In light of these considerations, AI should currently be viewed as a decision-support tool rather than a substitute for expert judgment.

## Figures and Tables

**Table 1:** Summary of the clinical applications of 3D aortic segmentation (EVAR: endovascular aneurysm repair, FEVAR: fenestrated endovascular aneurysm repair; BEVAR: branched endovascular aneurysm repair; ILT: intraluminal thrombus; PVAT: perivascular adipose tissue; FAI: fat attenuation index; CFD: computational fluid dynamics; FEA: finite element analysis; PMEG: physician-modified endograft).

Clinical Applications	Quantitative Features Analyzed
Preoperative planning (EVAR, FEVAR, BEVAR)	Precise measurements of aortic diameters, lengths, tortuosity, landing zones, and spatial orientation of branch vessels
Assessment of AAA progression and rupture risk prediction	Aneurysm sac volume, ILT volume, PVAT density, and morphometric changes
Post-EVAR surveillance: potential support for graft, lumen, aneurysm-sac, and endoleak assessment; endoleak detection requires appropriate delayed or multiphasic CTA and dedicated clinical validation.	Aortic remodeling, sac shrinkage, ILT and PVAT density (FAI) changes, endograft position, and possible endoleak-related changes
Patient-specific 3D models (CFD, FEA) for training, preoperative simulations, PMEG	Hemodynamic and biomechanical parameters, including wall shear stress, pressure distribution, and flow patterns

**Table 2:** Overview of the principal technical architectures, AI frameworks, and clinical-operational models currently used or proposed for aortic CTA segmentation, endovascular planning, and postoperative follow-up. (AI: artificial intelligence; CTA: computed tomography angiography; CFD: computational fluid dynamics; FEA: finite element analysis; Grad-CAM: Gradient-weighted Class Activation Mapping; XAI: Explainable Artificial Intelligence).

Technical Architectures	Clinical-Operational/Application Models
**Machine learning models**: broader category of algorithms that learn from data	**Manual segmentation**: segmentation performed entirely by the operator
**Deep learning models**: broad category including multilayer neural networks. The most commonly used are listed below:	**Semi-automatic segmentation**: the operator provides an initial input and the software performs part of the segmentation
**CNNs**: Convolutional Neural Networks	**Fully automatic segmentation**: the software generates segmentation masks without direct initial user intervention
2. **U-Net**: CNN-based architecture for biomedical image segmentation	**AI-assisted presegmentation**: AI generates preliminary segmentation masks that are subsequently reviewed
3. **U-Net++**: U-Net variant	**Human-in-the-loop AI-assisted segmentation**: AI output is reviewed, corrected, and validated by a vascular specialist or radiologist
4. **Attention U-Net**: U-Net variant incorporating attention mechanisms	**End-to-end workflow**: pipeline from raw CTA data to segmentation, centerline extraction and 3D modeling
5. **ResU-Net**: U-Net variant incorporating residual connections	**Digital twin**: patient-specific digital replica used for predictive simulation
6. **nnU-Net**: self-configuring framework for biomedical segmentation tasks	**Predictive simulation model**: CFD simulations and FEA used to estimate patient-specific hemodynamic and biomechanical parameters.
**Foundation models**: large-scale pre-trained models adaptable through transfer learning. Currently at an early stage of application.	**Radiomics/morphomics model**: extraction of quantitative imaging biomarkers from CTA
**XAI methods**: saliency maps, Grad-CAM, and heatmaps. Currently at an early stage of application.	**Clinical decision-support tool**: AI-based tools to support expert clinical judgment

## Data Availability

This narrative review synthesizes published literature. Figure 1 and Figure 3 include illustrative clinical CTA images and derived annotations or segmentations from our institution. The underlying clinical imaging data are not publicly available to protect patient privacy.

## Supplementary Materials

The following supporting information can be downloaded at: https://www.mdpi.com/article/10.3390/jimaging12100479/s1, Table S1: Characteristics and reported performance of primary studies on aortic CT segmentation. Studies are listed alphabetically by first author. NA: not available or not applicable, including information not reported or not reliably ascertainable from the available full text or other accessible sources. (AAA: abdominal aortic aneurysm; TAA: thoracic aortic aneurysm; ILT: intraluminal thrombus; PVAT: perivascular adipose tissue; CT: computed tomography; CTA: computed tomography angiography; CV: cross-validation; ASD: average surface distance; ASSD: average symmetric surface distance; HD: Hausdorff distance; HD95: 95th-percentile Hausdorff distance; NSD: normalized surface Dice; MIoU: mean intersection over union; LoA: limits of agreement; SD: standard deviation. Values are presented as mean ± SD unless otherwise specified); Figure S1: Reported Mean Dice coefficients by study and target structure. Points show mean Dice coefficient values from nine studies in Table S1; studies are ordered alphabetically within each target group. The labels give the number of evaluated CT scans (n), which does not necessarily equal the number of patients. Marker styles distinguish internal testing, internal validation, external evaluation, cross-validation, and an unascertainable internal/external status (NA). Dispersion measures and further methodological details are provided in Table S1. No pooled estimates, study weighting, or statistical comparisons are presented. *External evaluation indicates the classification reported by the original study; definitions of external val-idation were not uniform across studies. File S1: Scale for the Assessment of Narrative Review Articles – SANRA.

## Abbreviations

The following abbreviations are used in this manuscript:

3D	Three-dimensional
CTA	Computed Tomography Angiography
AI	Artificial Intelligence
EVAR	Endovascular Aneurysm Repair
AAA	Abdominal Aortic Aneurysm(s)
CT	Computed Tomography
ESVS	European Society for Vascular Surgery
DL	Deep Learning
MeSH	Medical Subject Headings
CNN	Convolutional Neural Network
FEVAR	Fenestrated Endovascular Aneurysm Repair
BEVAR	Branched Endovascular Aneurysm Repair
TEVAR	Thoracic Endovascular Aortic Repair
CFD	Computational Fluid Dynamics
MRI	Magnetic Resonance Imaging
DICOM	Digital Imaging and Communications in Medicine
HU	Hounsfield Units
ILT	Intraluminal Thrombus
MPR	Multiplanar Reconstruction
MIP	Maximum Intensity Projection
VR	Volume Rendering
FEA	Finite Element Analysis
TL	True Lumen
FL	False Lumen
PVAT	Perivascular Adipose Tissue
FAI	Fat Attenuation Index
ROI	Region of Interest
PACS	Picture Archiving and Communication System
ML	Machine Learning
ANNs	Artificial Neural Networks
XAI	Explainable Artificial Intelligence
WSS	Wall Shear Stress
DSC	Dice similarity coefficient
PMEG	Physician-modified endograft
