# Supplementary material for: Artificial Intelligence in 3D Aortic CTA Segmentation for Abdominal Aortic Aneurysm Management: A Narrative Review
Source: J Imaging. 2026 Oct 2;12(10):479. doi: 10.3390/jimaging12100479 (PMC13706595; doi:10.3390/jimaging12100479)
Supplement: Supplementary file 1 [file jimaging-12-00479-s001.zip › jimaging-4566869 - Figure S1 and Table S1.pdf]

Supplementary Materials: Artificial Intelligence in 3D Aortic CTA Segmentation for Abdominal Aortic Aneurysm Management: A Narrative Review

Leonardo Pasquetti *, Edoardo Pasqui, Eleonora Cerbini, Manfredi Giuseppe Anzaldi, Giovanna Grasso, Bruno Gargiulo, Giuseppe Alba and Gianmarco de Donato

Table S1. Characteristics and reported performance of primary studies on aortic CT segmentation. Studies are listed alphabetically by first author. NA: not available or not applicable, including information not reported or not reliably ascertainable from the available full text or other accessible sources. (AAA: abdominal aortic aneurysm; TAA: thoracic aortic aneurysm; ILT: intraluminal thrombus; PVAT: perivascular adipose tissue; CT: computed tomography; CTA: computed tomography angiography; CV: cross-validation; ASD: average surface distance; ASSD: average symmetric surface distance; HD: Hausdorff distance; HD95: 95th-percentile Hausdorff distance; NSD: normalized surface Dice; MIoU: mean intersection over union; LoA: limits of agreement; SD: standard deviation. Values are presented as mean ± SD unless otherwise specified).

Author and year	Study design	Patients and CT/CTA volumes	Training / validation / test and external status	Aortic segment	Target structures	Architecture or method	Dice	Boundary metric	Clinical quantity error	Data and code availability
**Bonechi et al. 2021**	Retrospective; single-centre	154 contrast-enhanced CT; patient count NA	Training: 134<br>Validation: 10<br>Test: 10<br>Internal test: Yes<br>External test: No	Aorta; extent NA	Binary aortic mask	2D U-Net / LinkNet; selected LinkNet with Inception-ResNet-v2	NA: MIoU: axial 83.45%; coronal 77.11%; sagittal 76.75%	NA	NA	Data: NA;<br>Code: NA

 https://doi.org/10.3390/jimaging12100479

Author and year	Study design	Patients and CT/CTA volumes	Training / validation / test and external status	Aortic segment	Target structures	Architecture or method	Dice	Boundary metric	Clinical quantity error	Data and code availability
**Caradu et al. 2021**	Retrospective; single-centre	100 patients / preoperative CTA; 38 separate training CTA	Training: 38<br>Validation: NA<br>Test: 100 (separate from training)<br>Internal test: NA<br>External test: NA	Infrarenal aorta; lowest renal artery to aortic bifurcation	Global aneurysm volume, lumen and ILT	PRAEVAorta; U-Net-like convolutional neural networks	Global: 0.95 ± 0.01<br>Lumen: 0.93 ± 0.05<br>ILT: 0.81 ± 0.10	Mean HD/slice (mm)<br>Global: 4.14 ± 6.20<br>Lumen: 4.61 ± 7.26<br>ILT: 4.76 ± 5.55	Mean absolute maximum-diameter error: 2.3 ± 2.1 mm versus senior surgeon	Data: NA;<br>Code: NA

https://doi.org/10.3390/jimaging12100479

**Author and year**	**Study design**	**Patients and CT/CTA volumes**	**Training / validation / test and external status**	**Aortic segment**	**Target structures**	**Architecture or method**	**Dice**	**Boundary metric**	**Clinical quantity error**	**Data and code availability**
**Derycke et al. 2025**	Retrospective; multicentre	1,000 pretraining CT; 280 fine-tuning CTA; 48 test CTA;	Training: 280 fine-tuning<br>Validation: 5-fold CV<br>Test: 48<br>Internal test: No separate set<br>External test: Yes	Aorto-iliac and proximal branches	Lumen, ILT and calcium	3D nnU-Net v2.2; pretraining and fine-tuning	Lumen: 0.97 ± 0.01<br>ILT: 0.94 ± 0.05<br>Calcium: 0.87 ± 0.04	ASD (mm)<br>Lumen: 0.30 ± 0.15<br>ILT: 0.61 ± 0.72<br>Calcium: 0.28 ± 0.28	NA	Data: public pretraining, private annotations;<br>Code: NA
**Fantazzini et al. 2020**	Retrospective; single-centre	80 preoperative CTA from AAA patients	Training: 64<br>Validation: 6<br>Test: 10<br>Internal test: Yes<br>External test: No	Thoracic and abdominal aorta, common iliacs and proximal branches	Aortic lumen	Coarse 2D U-Net plus three-view 2D U-Nets; multi-view fusion	0.928 ± 0.013	Symmetric surface distance (mm)<br>Mean: 0.711 ± 0.644<br>Maximum: 50.059 ± 37.285	NA	Data: NA;<br>Code: NA
**Ginzburg et al. 2024**	Retrospective; single-centre	200 patients / contrast-enhanced CT; 100 AAA and 100 controls	Training: 180 development<br>Validation: 5-fold CV<br>Test: 20<br>Internal test: Yes<br>External test: No	Right coronary origin to aortic bifurcation	Aorta and AAA extent; derived PVAT region	3D nnU-Net; geometric partitioning and PVAT extraction	Aorta: 0.977 ± 0.016<br>AAA: 0.972 ± 0.021	NA	AAA endpoint error (mm)<br>Inferior: 9.01 ± 13.41<br>Superior: 8.34 ± 7.19	Data: on request;<br>Code: Yes, public nnU-Net and PVAT analysis

https://doi.org/10.3390/jimaging12100479

Author and year	Study design	Patients and CT/CTA volumes	Training / validation / test and external status	Aortic segment	Target structures	Architecture or method	Dice	Boundary metric	Clinical quantity error	Data and code availability
**Mavridis et al. 2024**	Retrospective; multi-centre public data and single-centre AAA data	76 CT; 56 public non-AAA and 20 private AAA; patient total NA	Training: 80% public<br>Validation: 2-fold AAA<br>Test: 20% public<br>Internal test: Yes<br>External test: NA; AAA data used in tuning	Abdominal aorta	Binary aortic mask	3D U-Net<br>UNETR<br>SwinUNETR<br>SegResNet	AAA cohort<br>U-Net: 0.89 ± 0.07<br>UNETR: 0.80 ± 0.13<br>Swin: 0.85 ± 0.09<br>SegResNet: 0.81 ± 0.13	ASSD:<br>0.04 ± 0.02;<br>0.05 ± 0.02;<br>0.05 ± 0.02;<br>0.06 ± 0.02	NA	Data: Yes, public datasets;<br>Code: NA
**Robbi et al. 2025**	Retrospective; public multicentre datasets	1,228 pretraining CT; 220 cropped CTA; 20 additional full-pipeline CTA; unique total NA	Training: 200 SegResNet<br>Validation: NA<br>Test: 20 + 20<br>Internal test: 20<br>External test: 20	Abdominal aorta, visceral branches and iliacs	AAA lumen, ILT, calcium, branches and sealing zones	BRAVE: nnU-Net + SegResNet; geometric and tissue analysis	Full pipeline<br>Lumen: 0.98 ± 0.01<br>ILT: 0.97 ± 0.03<br>Calcium: 0.60 ± 0.24	HD (mm)<br>Lumen: 5.34 ± 2.52<br>ILT: 4.52 ± 5.02<br>Calcium: 14.28 ± 7.50	AAA diameter bias: −0.37 mm (95% LoA −2.78 to 2.04)<br>Neck bias: 0.35 mm	Data: Yes, public sources;<br>Code: NA
**von Deylen et al. 2025**	Technical development; multicentre public dataset	56 AVT CTA; 13 additional external CTA for workflow evaluation; patient total NA	Training: 44<br>Validation: 6<br>Test: 6<br>Internal test: Yes<br>External test: Workflow only (13 CTA)	Aortic vessel tree	Aortic and branch lumen; surface and centreline	AortaAnalyzer; 3D ResU-Net	0.949 ± 0.014	HD95: 5.25 ± 5.73 mm	NA	Data: Yes, public datasets; external data restricted;<br>Code: NA; online platform available
**Wasserthal et al. 2023**	Retrospective; CT acquired at 8 sites	1,204 patients / CT; mixed contrast phases	Training: 1,082<br>Validation: 57<br>Test: 65 internal<br>Internal test: Yes<br>External test: No	Thoracic and abdominal aorta	Aorta among 104 structures	TotalSegmentator v1; nnU-Net	Aorta: 0.981	Aortic NSD: 0.997 (3-mm tolerance)	NA	Data: Yes, public annotated dataset (Zenodo);<br>Code/model: Yes, publicly developed model (GitHub)
**Weiss et al. 2026**	Retrospective; single-centre development; external multi-centre test	Internal: 110 patients / 220 paired CTA<br>External: 60 CTA<br>Total: 280 CTA	Training: 176 (88 patients)<br>Validation: 44 (22 patients)<br>Test: 60 external<br>Internal evaluation: Yes<br>External test: Yes	Infrarenal AAA; lowest renal artery to bifurcation	Aneurysm sac and lumen; ILT volume by subtraction	Modified nnU-Net; final configuration NA	Sac: 0.972 ± 0.013 internal; 0.960 ± 0.035 external<br>Lumen means: 0.963 internal; 0.941 external	Mean per-slice HD, internal / external (mm)<br>Sac: 2.053 / 4.524<br>Lumen: 1.772 / 1.797	Volume bias (mL)<br>Internal: sac −0.32; lumen −1.41; ILT +1.09<br>External: −2.68 to +1.10	Data: Yes, on request;<br>Code: Yes, public (GitHub)

https://doi.org/10.3390/jimaging12100479

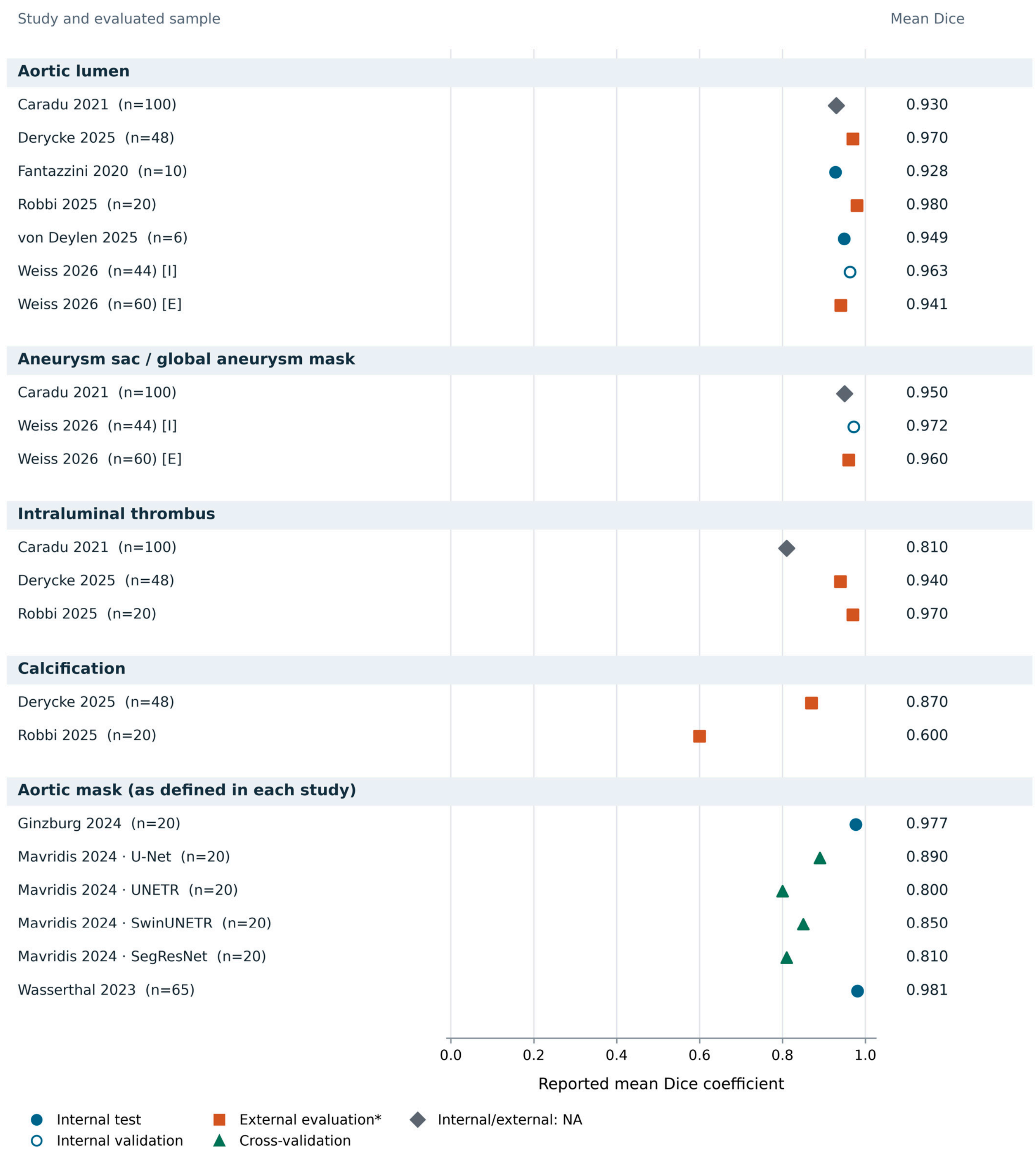

Figure S1. Reported Mean Dice coefficients by study and target structure. Points show mean Dice coefficient values from nine studies in Table 3; studies are ordered alphabetically within each target group. The labels give the number of evaluated CT scans (n), which does not necessarily equal the number of patients. Marker styles distinguish internal testing, internal validation, external evaluation, cross-validation, and an unascertainable internal/external status (NA). Dispersion measures and further methodological details are provided in Table 3. No pooled estimates, study weighting, or statistical comparisons are presented. *External evaluation indicates the classification reported by the original study; definitions of external validation were not uniform across studies.

https://doi.org/10.3390/jimaging12100479
